# Valgus Rotational Dome Osteotomy for Neglected Adult Blount’s Disease: A Surgical Case Report

**DOI:** 10.7759/cureus.84764

**Published:** 2025-05-25

**Authors:** Felix Rivera Troia, Carlos J Perez Lopez

**Affiliations:** 1 Orthopedic Surgery, Ponce Health Sciences University, Ponce, PRI

**Keywords:** blount's disease, dome osteotomy, medial compartment varus overload, orthopedic intervention, tibia vara

## Abstract

Blount’s disease (BD) is a progressive lower extremity deformity resulting from impaired growth of the proximal medial tibial physis, commonly leading to tibia vara. While early cases may benefit from conservative management, surgical correction is often required in neglected or severe presentations, particularly in adults. We report the case of an adult female with an eight-year history of medial-sided knee pain secondary to medial compartment varus overload as a result of tibia vara due to neglected BD. Following significant weight loss, the patient underwent a valgus-producing rotational dome tibial osteotomy with plate fixation of the medial tibial plateau, achieving 15 degrees of angular correction in a single procedure. Given the high bone contact of both sides of the osteotomy, the patient had a fast and successful recovery. At the final follow-up, six years after the procedure, the patient demonstrated proper limb alignment, resolution of pain, and return to daily physical activities without complications. This case highlights the viability of this surgical technique as a potential option for managing severe tibia vara deformities in adult patients with neglected BD.

## Introduction

Pathological tibia vara deformity in skeletally immature individuals, otherwise known as Blount’s disease (BD), is a progressive abnormality affecting the proximal medial tibial physis and epiphysis that results in a cross-sectional deformity with pronounced tibial varus [1]. The condition affects less than one percent of the United States population and is more prevalent in males than females [2]. The exact pathophysiology of BD remains uncertain. However, some researchers propose that excessive medial compartment overload on the proximal tibial physis disrupts native cartilage growth, leading to altered endochondral bone formation [2]. Additionally, Montgomery et al. suggest that factors such as elevated body mass index and low vitamin D levels significantly contribute to the development of this deformity [3].

BD is classified into infantile, juvenile, and adolescent forms based on age of onset and radiological characteristics, with infantile and adolescent types being the most commonly described in the literature. The infantile form, which presents before the age of 10, is often bilateral and commonly affects overweight Black children of African descent, with a particularly high prevalence in the Caribbean population [4]. This form is considered to be the most alarming as it has the potential to quickly progress to severe disease manifestations such as knee instability and early fusion of the tibial physis that can lead to limb length discrepancies and tibial procurvatum deformities, among others [5]. The adolescent form typically occurs in older children and is usually unilateral, though it can occasionally affect both limbs [2].

The management of BD varies on a case-by-case basis. While some authors advocate for the use of conservative approaches such as bracing [6,7], others report successful outcomes with surgical interventions [8-10]. Although not commonly performed, dome tibial osteotomies have been described as a treatment method that has shown promising clinical results for the correction of these deformities [11-13]. We present the case of an adult patient with an eight-year history of knee pain due to neglected BD who underwent surgical correction for a severe tibia vara deformity using the valgus-producing rotational dome tibial osteotomy technique. This report focuses on the surgical technique employed and patient-reported outcomes years after the procedure.

## Case presentation

This is the case of a 24-year-old female with a previous diagnosis of BD who arrived at the clinic due to left knee pain. The pain was described as constant and localized to the medial side of the knee, reaching a pain scale of 8/10 with loading activities such as simple mall walking. Eight years prior, she was evaluated by a pediatric specialist who diagnosed her with the condition, provided guidance, and discussed the possibility of corrective surgery. However, at the time, she declined any invasive intervention due to the absence of symptoms. Nevertheless, as she grew older, the deformity progressed, resulting in intolerable knee pain and subsequent referral to the clinic.

On observation, the patient had an obese body habitus, ambulated independently with a mild varus thrust gait, and exhibited a marked varus deformity of the left lower extremity. Physical examination did not demonstrate other deformities such as in the spine, pelvis, hips, ankles, or feet. Additionally, she demonstrated an adequate range of motion at the hip, knee, and ankle joints of the affected extremity. At this visit, the patient was further counseled on her condition and the possibility of undergoing corrective surgery and was advised to return to the clinic with long cassette standing anteroposterior radiographs of the lower extremities for further assessment.

**Figure 1:**
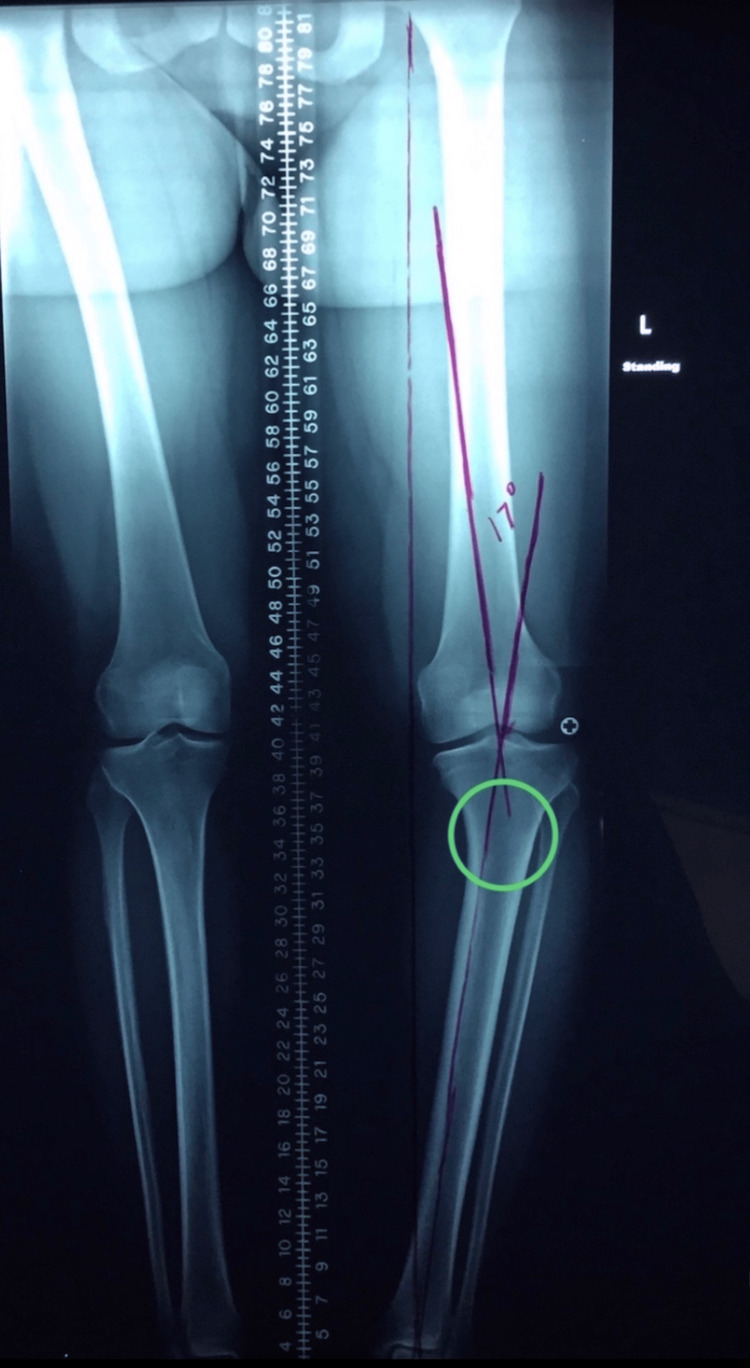
Long-cassette standing anteroposterior radiograph of the lower extremities demonstrating severe tibia vara deformity of the left lower extremity with a target correction angle of 17 degrees planned. Target correction angle to bring the deformity down to neutral was calculated by intersecting lines, one drawn from the center of the femoral head to the lateral aspect of the tibial spine and another from the center of the tibial plafond to the lateral tibial spine. The angle created by the intersection of these two lines is the resultant target correction angle. Green circle identifying the site for the osteotomy cut.

At her follow-up visit, the patient returned with the requested imaging, which revealed a severe tibia vara deformity of the left lower extremity with a target correction angle of 17 degrees (Figure 1). A detailed discussion on corrective surgery was held, but the patient chose to postpone the procedure for a year due to personal reasons. She was advised to focus on losing weight during this time and to return when ready for surgery.

At the one-year follow-up, the patient's knee condition remained unchanged. However, she had lost 123 lbs and expressed readiness for surgery. Subsequently, a valgus-producing rotational dome tibial osteotomy was planned for a 17-degree correction of a severe tibia vara deformity.

Surgical technique

Operative consent was obtained, and the patient was taken to the operating room. She was placed in the supine position with all bony prominences well-padded. The left knee was examined under general anesthesia, and the site was prepped and draped in the usual sterile manner.

Anteromedial and anterolateral portals were established, and a diagnostic arthroscopy was performed, which revealed scattered grade 2 chondromalacia in the medial femoral condyle and medial tibial plateau and a complex degenerative tear of the medial meniscus with a predominant horizontal cleavage pattern. A partial meniscectomy was performed on the posterior horn of the medial meniscus, leaving it back on a stable rim. Subsequently, the joint was copiously irrigated and drained.

**Figure 2:**
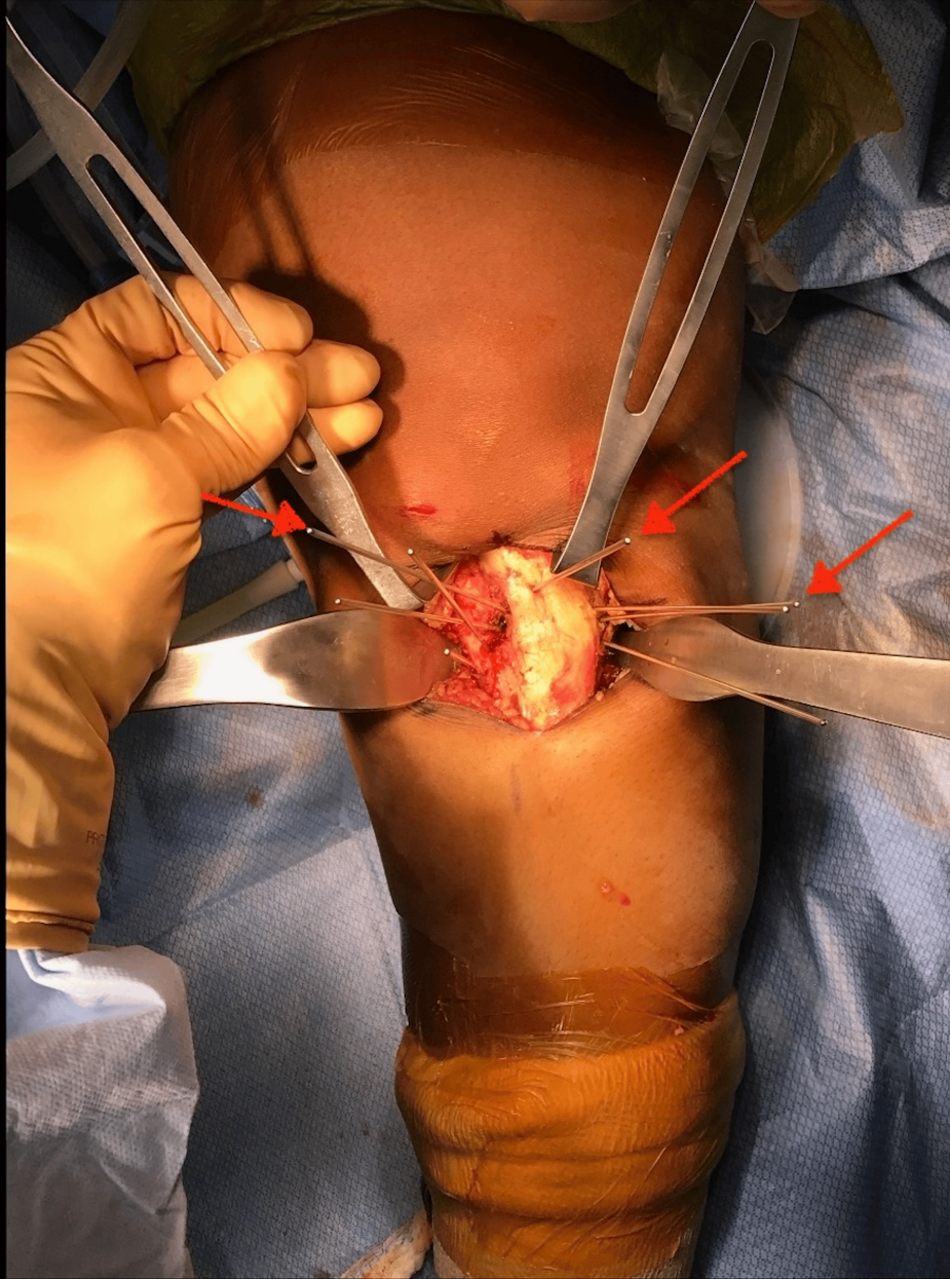
Guide pins placed (red arrows) on the proximal tibia and positioned in a crescent shape to guide the saw for the osteotomy cut around the patellar tendon.

**Figure 3:**
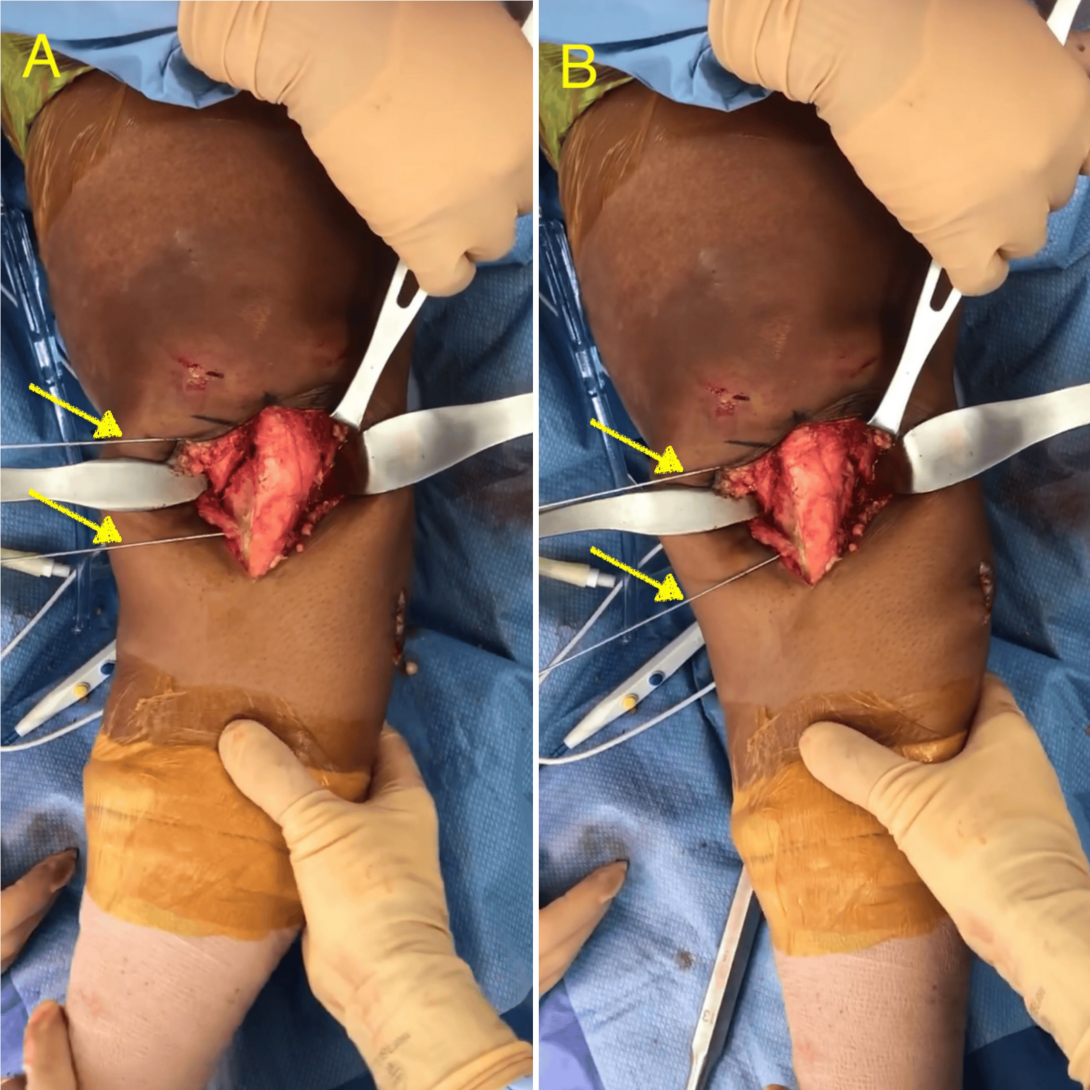
Baseline alignment of the patient's left lower extremity following the osteotomy cut (A) and extremity in neutral alignment after correction of 15 degrees of tibia vara deformity (B). Parallel Kirschner wire pins (yellow arrows) were placed to measure the correction after osteotomy.

**Figure 4:**
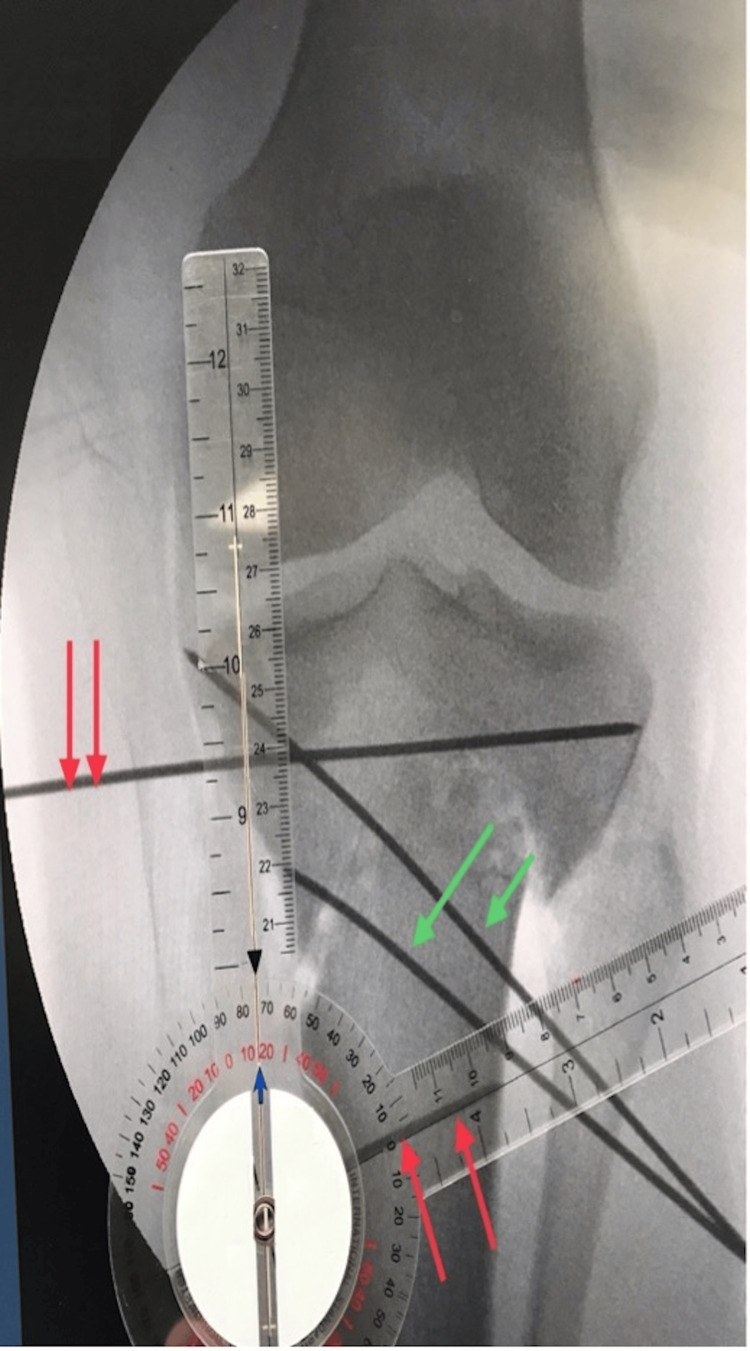
Fluoroscopy image demonstrating intraoperative assessment of the correction. Parallel Kirschner wires after correction measure 75 degrees from each other (red arrows), demonstrating 15 degrees of correction. Temporary fixation with Kirschner wires prior to definitive plate fixation (green arrows).

Following the arthroscopy, the knee was re-prepped with ChloraPrep™ (Becton, Dickinson and Company, Franklin, NJ, USA), and a tourniquet was inflated. A midline anterior incision in the proximal tibia was performed, the tibia was exposed, and the patellar tendon insertion to the tibial tubercle was preserved. A dome osteotomy was carried out by connecting with an osteotome multiple drill holes (Figure 2). An accessory lateral incision was performed to expose and osteotomize the fibula to allow adequate tibial correction. A correction of 15 degrees was achieved (Figures 3A-3B) and confirmed by fluoroscopy (Figure 4) with a plumb line from the hip to the ankle. Multiple fluoroscopic views were viewed to ensure proper alignment. Plate fixation was performed on the medial tibia with multiple locking and nonlocking screws. Very good fixation was obtained. Subsequently, the area was copiously irrigated, and the incision closed in layers.

**Figure 5:**
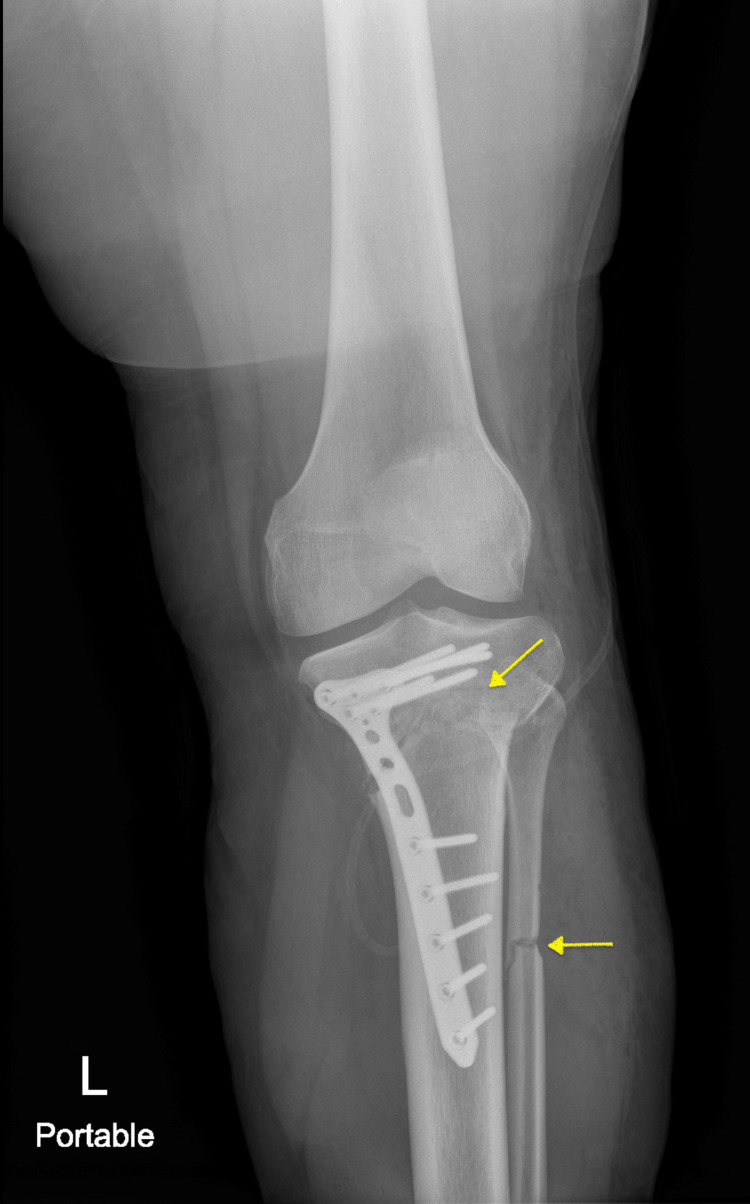
Follow-up radiograph with an anteroposterior view of the patient's left lower extremity at six weeks postoperatively demonstrating proper alignment and adequate healing osteotomy sites (yellow arrows).

Although a 17-degree correction was initially planned, a final correction of 15 degrees was performed intraoperatively to avoid valgus overcorrection, based on real-time alignment assessment. The bony overhang can occur during rotational osteotomy correction, but in this case, despite 15 degrees of correction, no significant overhang of bone was noticed after the correction. Postoperatively, she was instructed to start toe-touch weight bearing, progressing to partial weight bearing as tolerated by the end of the first month postoperatively, and was prescribed pain medication as needed. Early range of motion was encouraged, and the patient was allowed full weight-bearing six weeks after surgery. She was followed up at 1, 3, 6, and 12 weeks after surgery and was encouraged to do physical therapy and stretching exercises as tolerated. Over time, she demonstrated progressive improvement with no reported postoperative complications. Follow-up knee radiographs confirmed stable plate fixation of the proximal medial tibia with no signs of migration and a well-healing osteotomy site (Figure 5).

At a six-year follow-up, the patient maintained proper limb alignment and reported resolution of medial knee pain, with only occasional soreness after strenuous activity. She reported a satisfactory outcome, stating that she could perform gym workouts with only minimal soreness, noted only after heavy knee-loading activities or in cold weather.

## Discussion

BD is a developmental lower extremity deformity caused by excessive loading on the medial compartment of the proximal tibial physis, which disrupts normal endochondral bone formation [2]. Several studies have identified risk factors associated with the development of the disease, including individuals of African descent and Caribbean origin, as well as elevated body mass index [3,4]. Notably, the patient presented in this case encompasses all of these demographic characteristics. Furthermore, if neglected, BD can progress to permanent complications such as limb length discrepancies, abnormal gait patterns, and early-onset knee osteoarthritis [14].

Conservative management strategies are documented in the literature for treating Blount's [6,7]. For instance, a study by* *Richards et al.* *advocates for the use of bracing in cases of infantile BD. Their study found that bracing was effective in correcting unilateral deformities in most cases, often avoiding the need for surgical intervention. However, they noted that bracing was less effective in patients with bilateral disease, who frequently required subsequent osteotomy procedures for adequate correction [6]. Moreover, a study by de Miranda Luzo et al. further supports the use of conservative management through molded orthoses. Nevertheless, their findings indicated that the effectiveness of this approach is significantly reduced in patients over the age of three compared to younger individuals [7].

Several surgical techniques have been described for correcting tibia vara deformities [8-13,15]. The ‘V’ osteotomy technique described by* *Miraj et al. involves creating a triangular cut in the proximal tibia, excising the osteotomized bone, and internally rotating the proximal segment to form a V-shaped configuration that corrects the deformity [9]. Their study demonstrated effective angular correction postoperatively, while also allowing for early lower limb mobilization and weight-bearing, with minimal risk of recurrence at the one-year follow-up [9]. Furthermore,* *Karuppal et al. reported favorable outcomes using their novel ‘Z’ osteotomy technique. This approach involves performing osteotomies on both the tibia and fibula. The tibia is cut in a Z-shaped pattern, with a central wedge segment removed. The distal tibial segment is then derotated and laterally angulated to re-engage it in proper alignment, effectively correcting the deformity [10]. Both techniques yielded successful outcomes comparable to those observed in the patient presented in this case. Furthermore, although BD primarily involves angular deformities arising from disordered growth at the proximal tibial physis, clinicians should remain vigilant for atypical bone pathologies such as ectopic or heterotopic ossification, as highlighted in rare reports of extraskeletal bone formation in non-musculoskeletal sites [16], thus underscoring the broader spectrum of bone formation abnormalities.

The valgus-producing rotational dome tibial osteotomy resulted in successful correction and pain relief, consistent with previous studies reporting favorable alignment and functional outcomes using this method [11-13]. This method has been demonstrated to be useful, particularly in patients with multi-planar deformities [17]. The technique involves an anterior approach to the proximal tibia, with meticulous dissection carried down to the periosteum. The level of the osteotomy is determined to be just proximal to and around the patellar tendon in the metaphyseal bone. Next, a crescent-shaped plane is marked by guide pins to further delineate the osteotomy site, and a bone saw and osteotomes are used to cut the bone following this crescent plane. Once the osteotomy is complete, angular and rotational correction is achieved through serial intraoperative manipulation until target correction is attained [18]. The senior author of this report suggests that cutting around the tibial tubercle with the present technique avoids affecting the coronal knee joint line, as could potentially happen in other osteotomies with such a large correction angle.

Geith reported optimal clinical and radiographic results from correcting infantile tibia vara deformities using the dome osteotomy technique [11]. However, unlike our case, which required plate fixation of the medial tibial plateau, they used Kirschner wires and plaster molds to hold the osteotomy site in place. Moreover, Watanabe et al. reported similar outcomes in their study population, with the added benefit of early full weight-bearing following a corrective dome osteotomy combined with Ilizarov frame application for limb lengthening [12]. However, they suggest that while the technique was effective for alignment correction, it did not demonstrate any significant advantage for limb lengthening.

## Conclusions

This case highlights the successful application of a valgus-producing rotational dome tibial osteotomy technique with plate fixation employed for the correction of a severe tibia vara deformity in an adult female with neglected BD. At the six-year follow-up, the patient reported a favorable outcome, suggested by pain resolution, restored lower limb alignment, and a return to daily physical activity. Although this is based on a single case, these results suggest that valgus-producing dome osteotomy may be a viable option for correcting advanced angular and rotational deformities when conservative treatments have failed.
